# Supplementary figures and images for: Structural patterns for transmedia storytelling
Source: PLoS One. 2020 Jan 31;15(1):e0225910. doi: 10.1371/journal.pone.0225910 (PMC6993987; doi:10.1371/journal.pone.0225910)

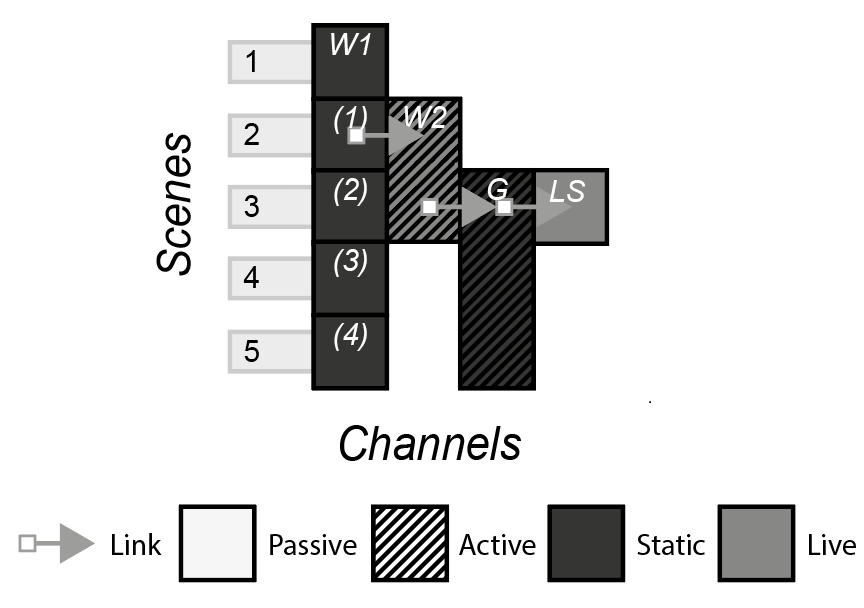

Supplement: S1 Fig — (PNG) [file pone.0225910.s001.png]

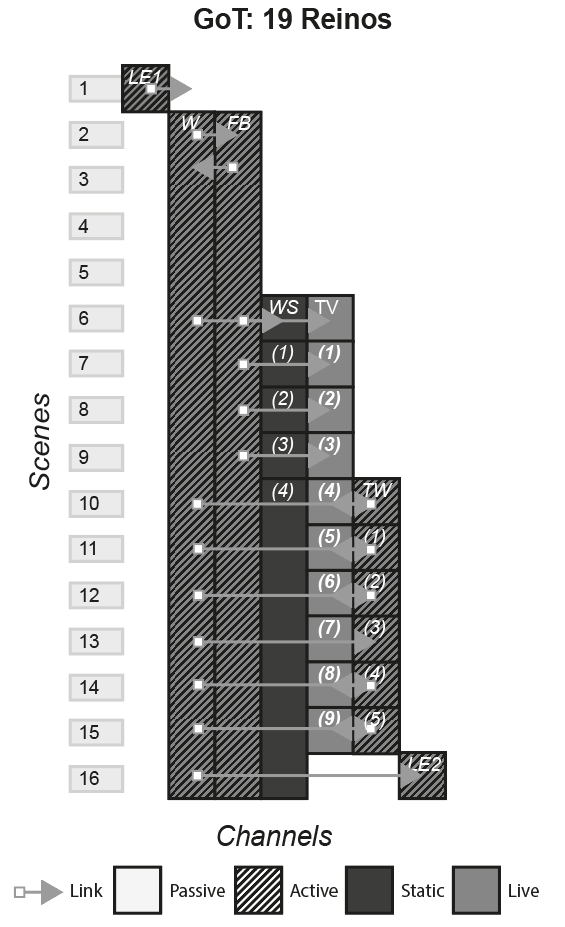

Supplement: S2 Fig — (PNG) [file pone.0225910.s002.png]

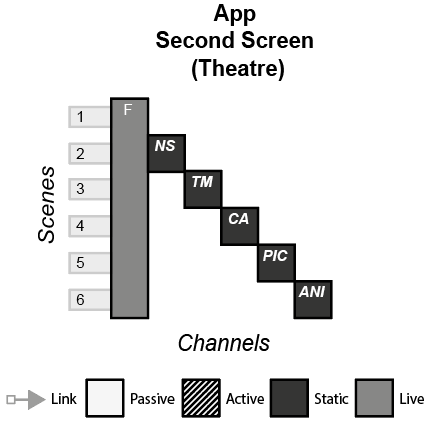

Supplement: S3 Fig — (PNG) [file pone.0225910.s003.png]

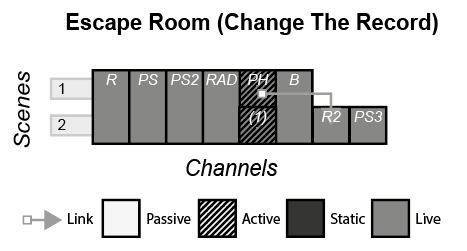

Supplement: S4 Fig — (PNG) [file pone.0225910.s004.png]

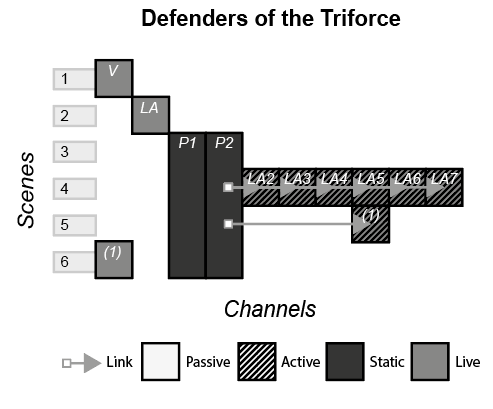

Supplement: S5 Fig — (PNG) [file pone.0225910.s005.png]

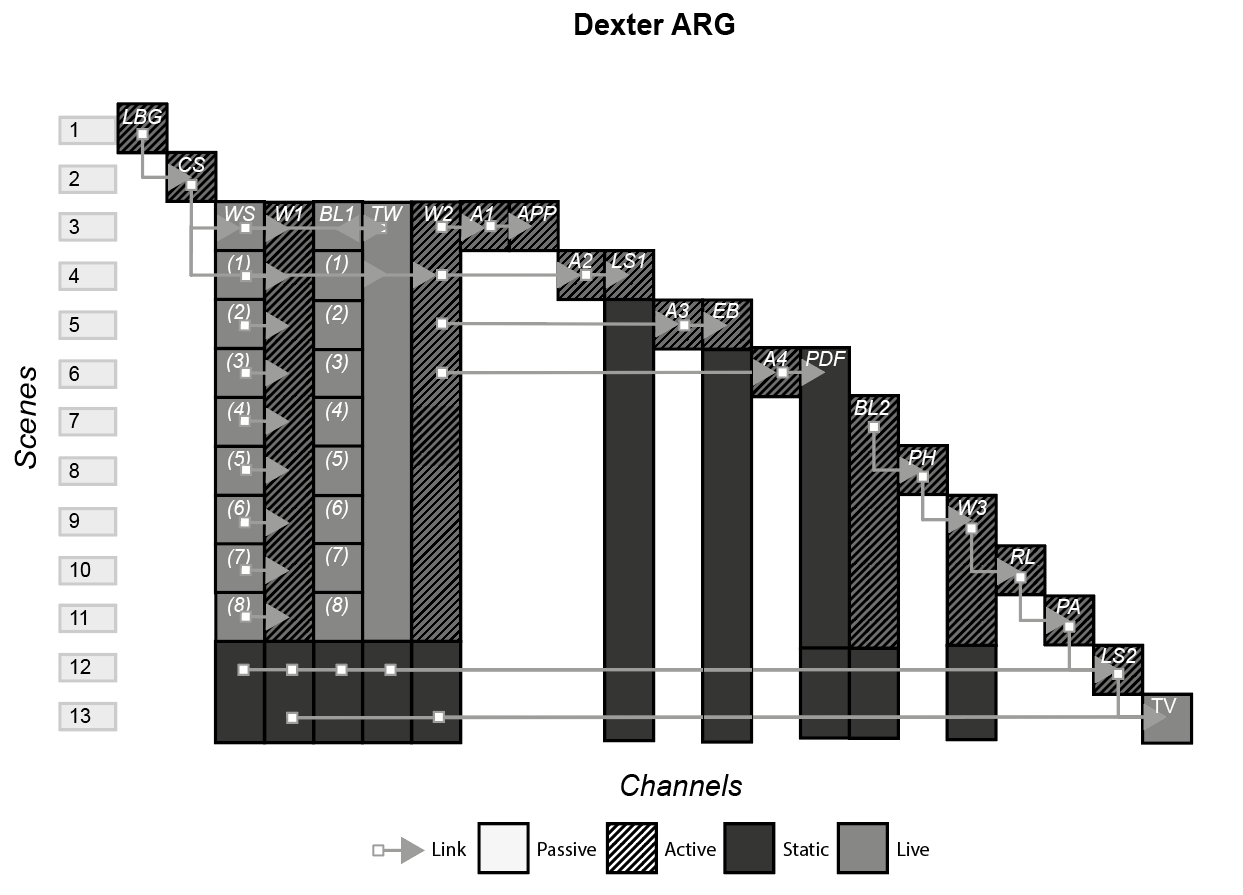

Supplement: S6 Fig — (PNG) [file pone.0225910.s006.png]

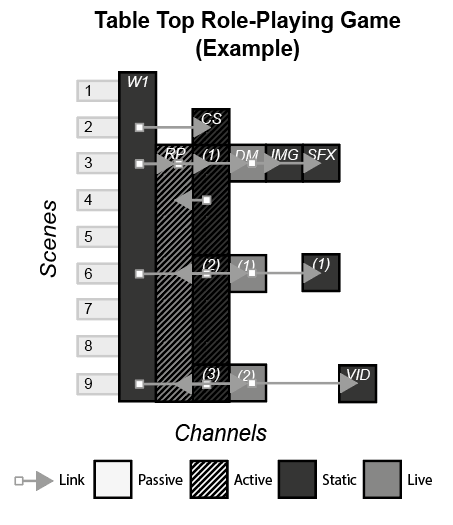

Supplement: S7 Fig — (PNG) [file pone.0225910.s007.png]

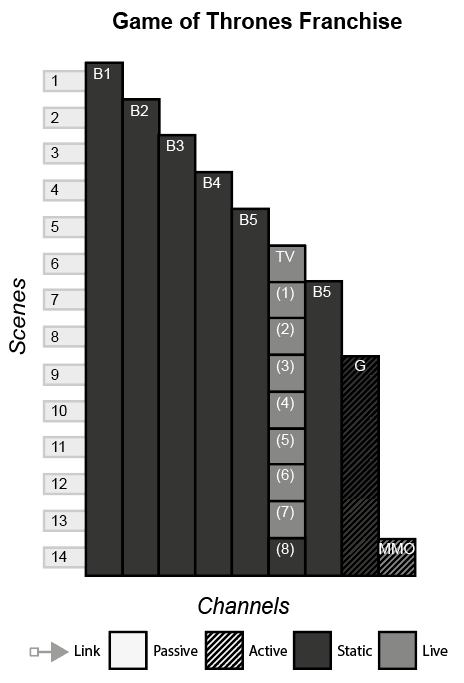

Supplement: S8 Fig — (PNG) [file pone.0225910.s008.png]

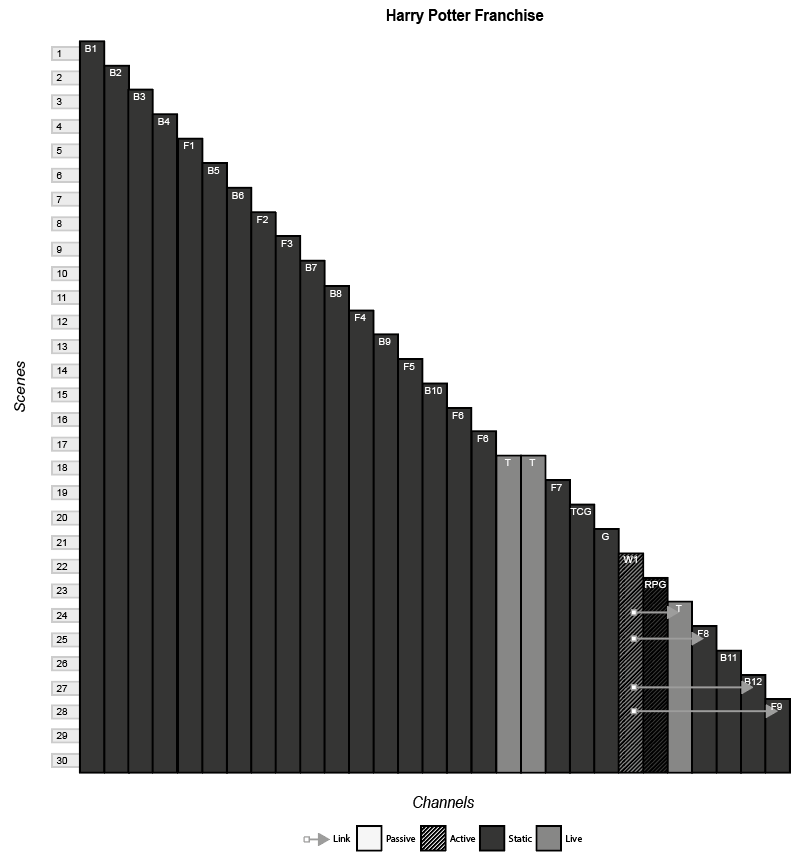

Supplement: S9 Fig — (PNG) [file pone.0225910.s009.png]

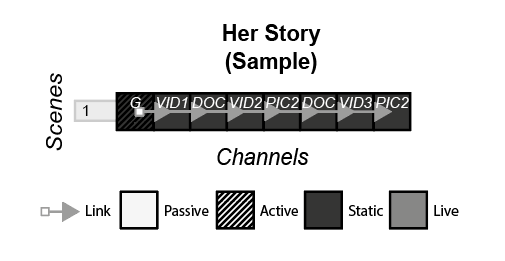

Supplement: S10 Fig — (PNG) [file pone.0225910.s010.png]

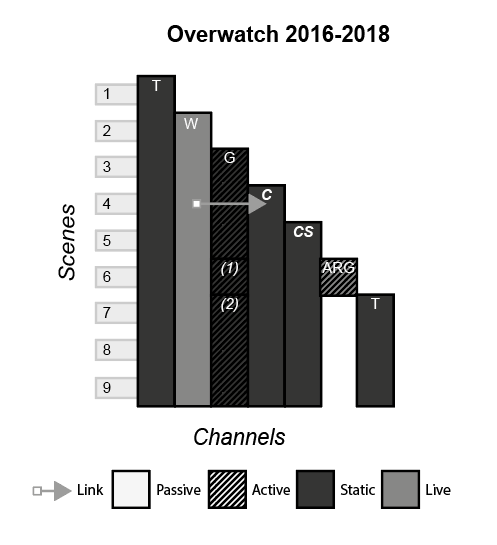

Supplement: S11 Fig — (PNG) [file pone.0225910.s011.png]

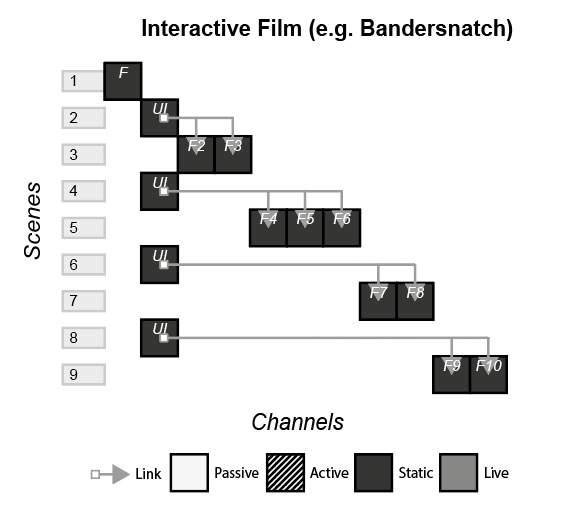

Supplement: S12 Fig — (PNG) [file pone.0225910.s012.png]

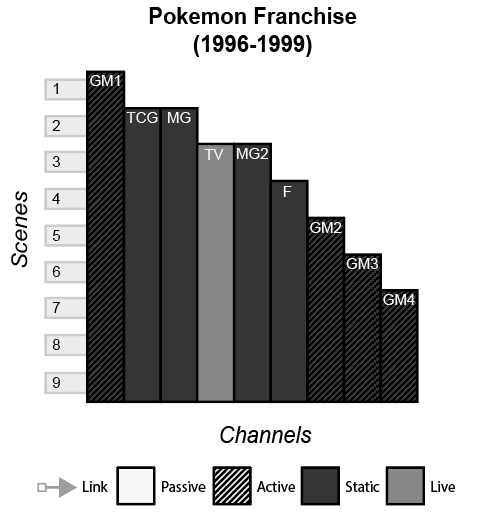

Supplement: S13 Fig — (PNG) [file pone.0225910.s013.png]

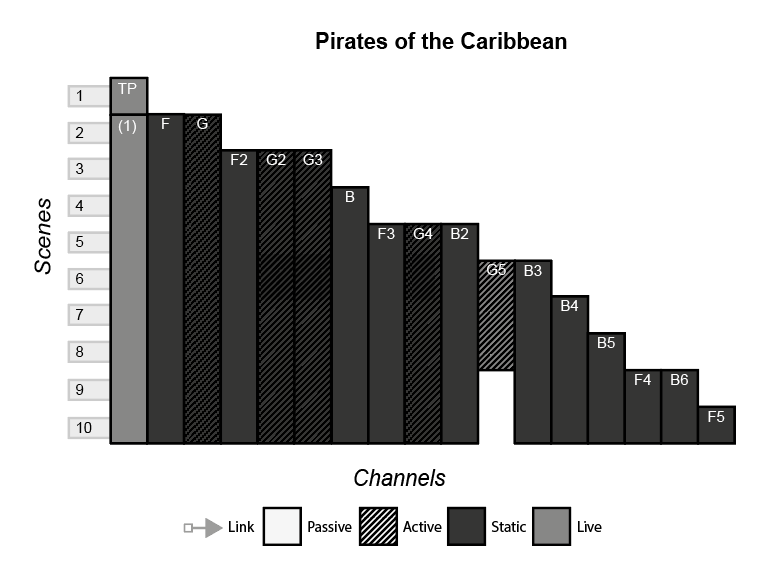

Supplement: S14 Fig — (PNG) [file pone.0225910.s014.png]

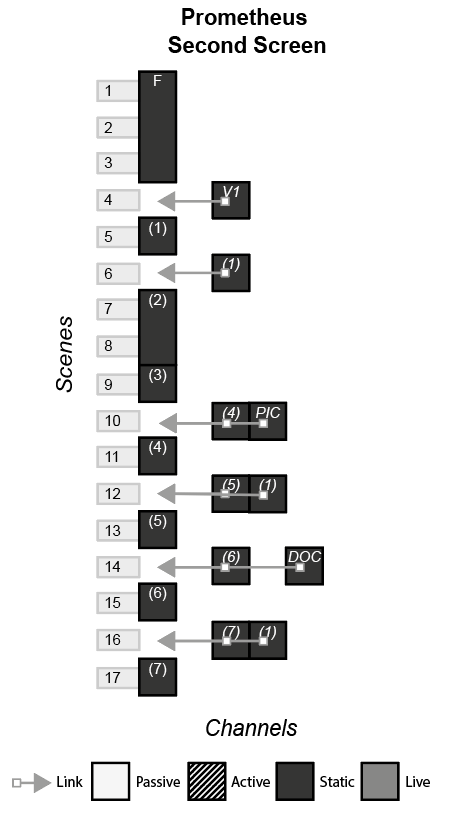

Supplement: S15 Fig — (PNG) [file pone.0225910.s015.png]

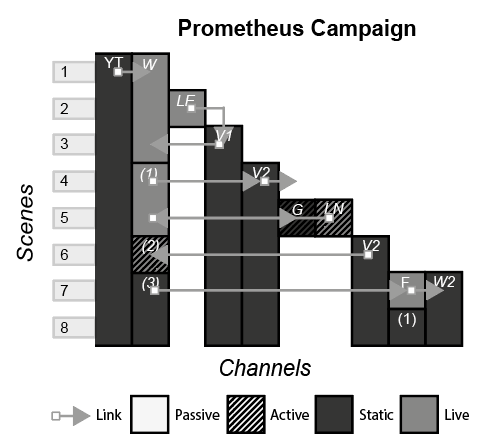

Supplement: S16 Fig — (PNG) [file pone.0225910.s016.png]

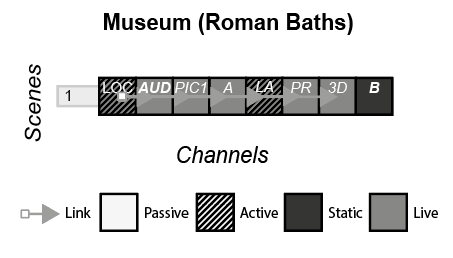

Supplement: S17 Fig — (PNG) [file pone.0225910.s017.png]

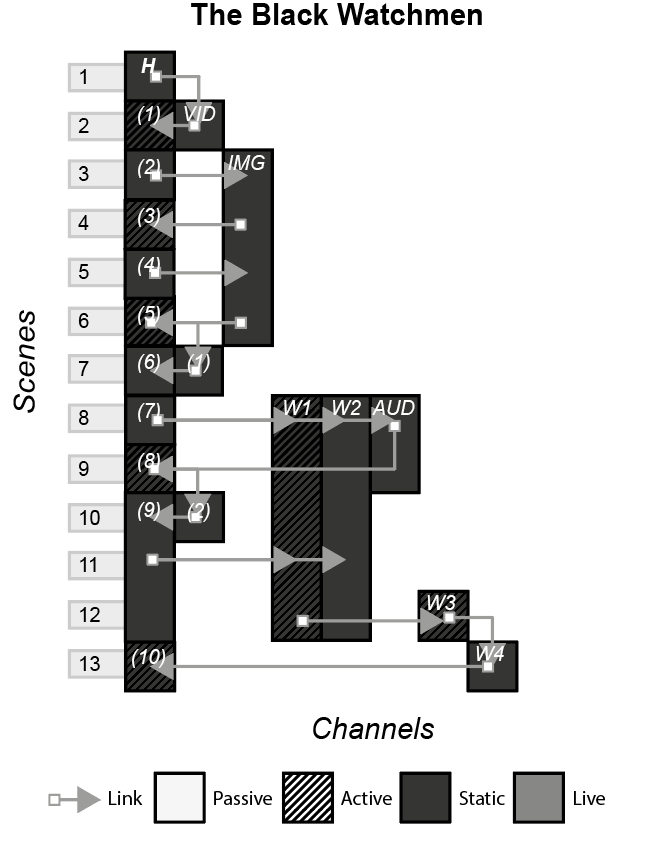

Supplement: S18 Fig — (PNG) [file pone.0225910.s018.png]

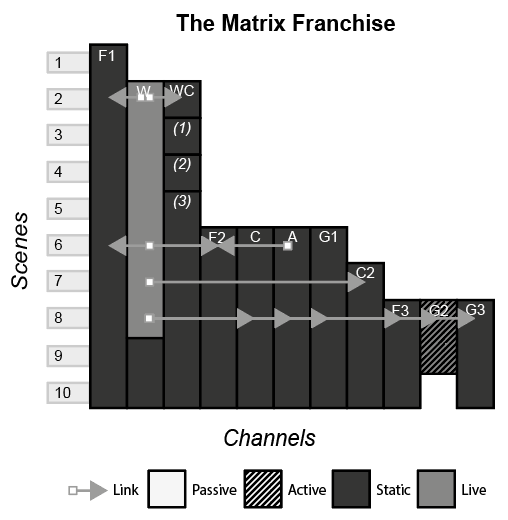

Supplement: S19 Fig — (PNG) [file pone.0225910.s019.png]

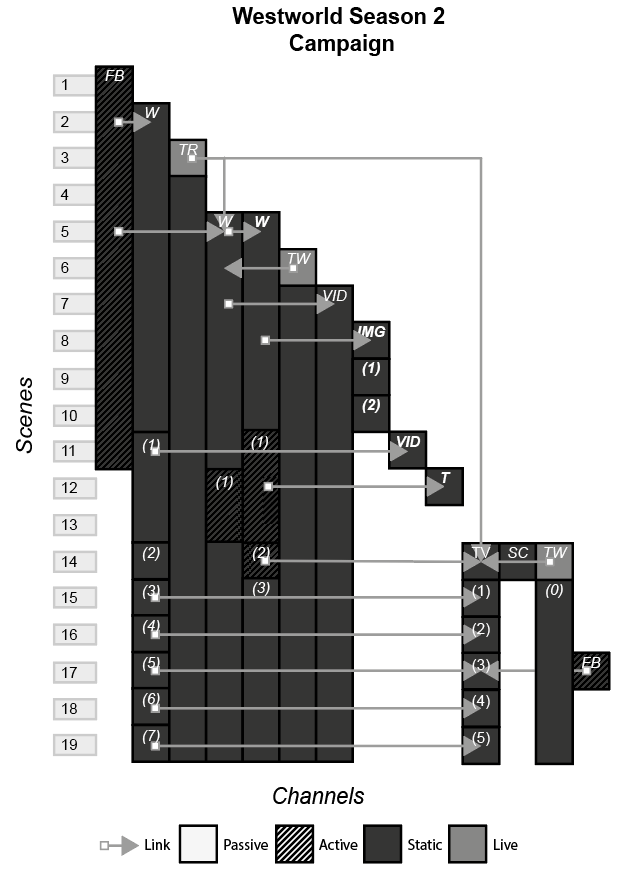

Supplement: S20 Fig — (PNG) [file pone.0225910.s020.png]

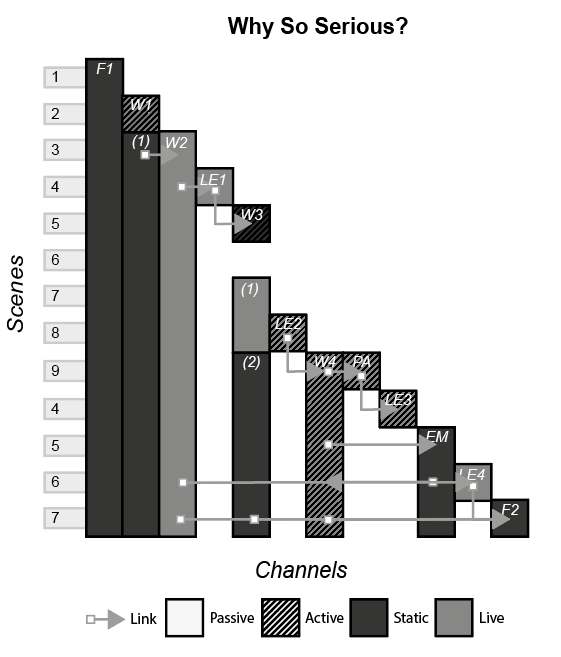

Supplement: S21 Fig — (PNG) [file pone.0225910.s021.png]

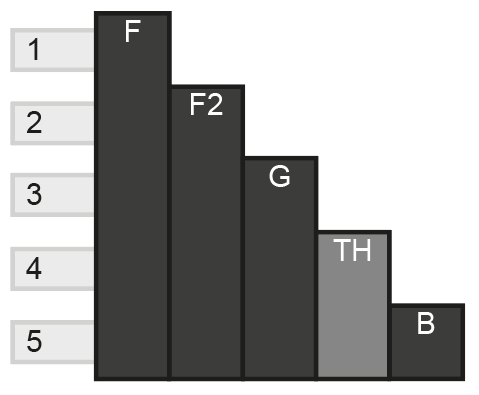

Supplement: S22 Fig — (PNG) [file pone.0225910.s022.png]

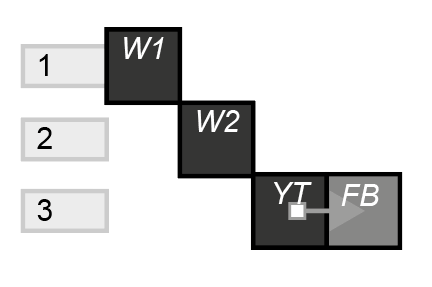

Supplement: S23 Fig — (PNG) [file pone.0225910.s023.png]

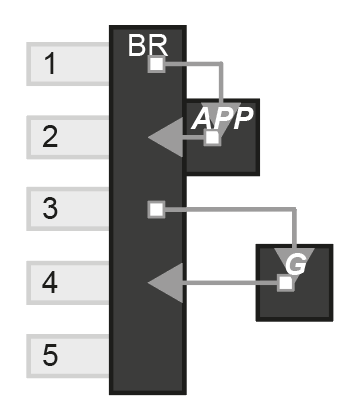

Supplement: S24 Fig — (PNG) [file pone.0225910.s024.png]

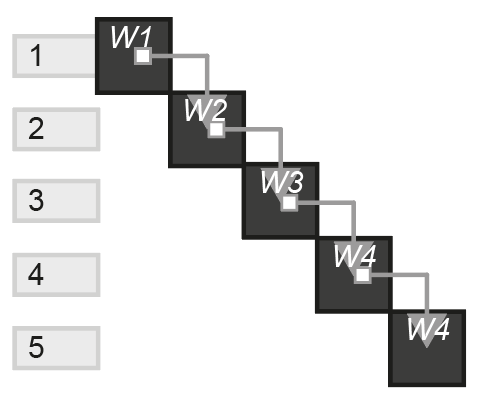

Supplement: S25 Fig — (PNG) [file pone.0225910.s025.png]

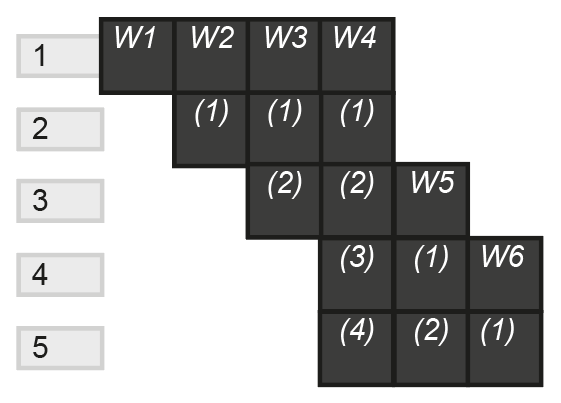

Supplement: S26 Fig — (PNG) [file pone.0225910.s026.png]

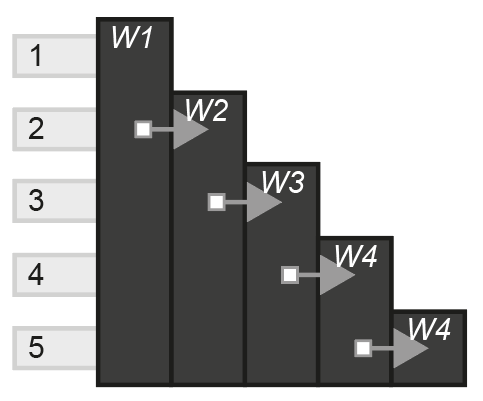

Supplement: S27 Fig — (PNG) [file pone.0225910.s027.png]

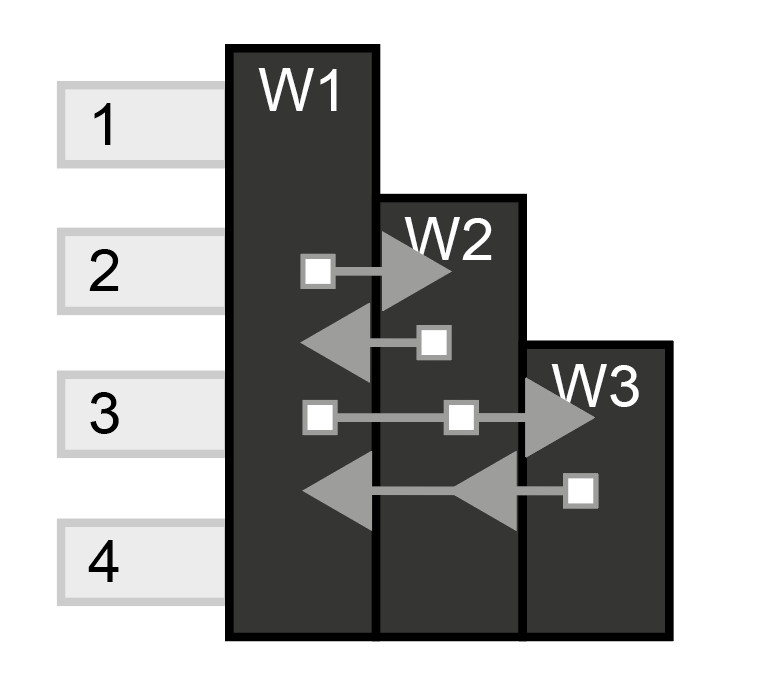

Supplement: S28 Fig — (PNG) [file pone.0225910.s028.png]

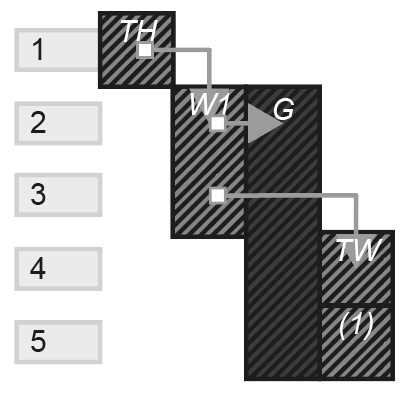

Supplement: S29 Fig — (PNG) [file pone.0225910.s029.png]

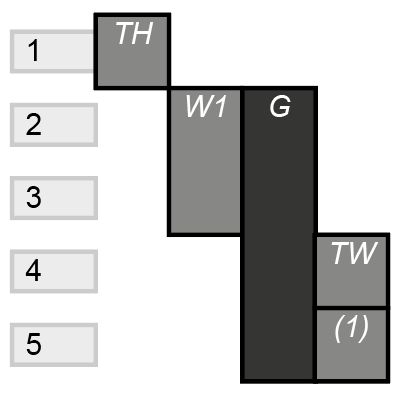

Supplement: S30 Fig — (PNG) [file pone.0225910.s030.png]

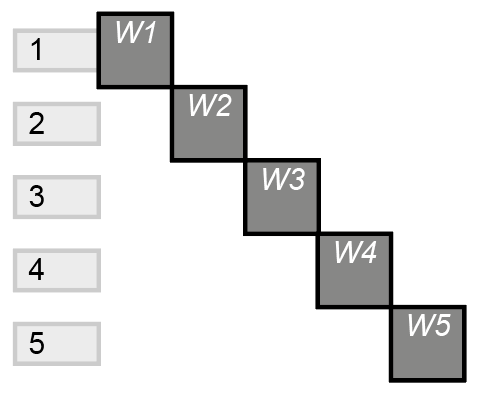

Supplement: S31 Fig — (PNG) [file pone.0225910.s031.png]

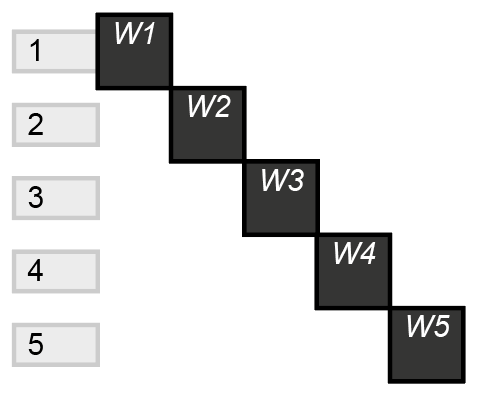

Supplement: S32 Fig — (PNG) [file pone.0225910.s032.png]

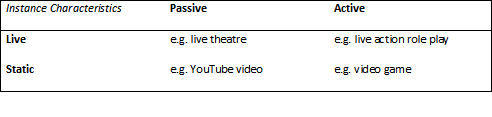

Supplement: S1 Table — (PNG) [file pone.0225910.s033.png]

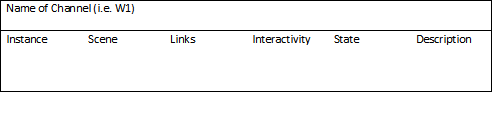

Supplement: S2 Table — (PNG) [file pone.0225910.s034.png]

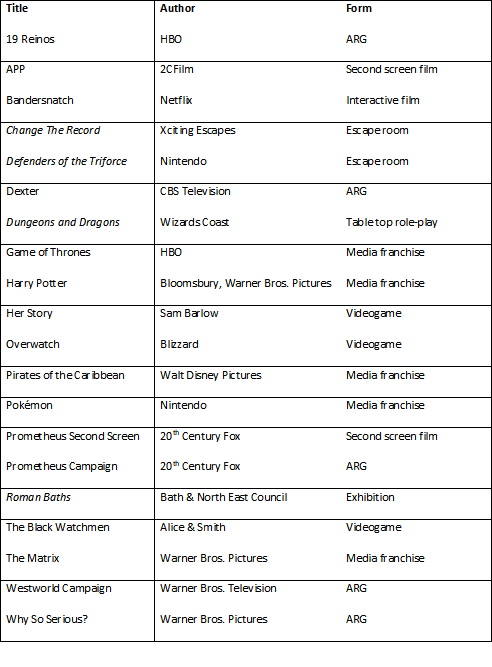

Supplement: S3 Table — (PNG) [file pone.0225910.s035.png]

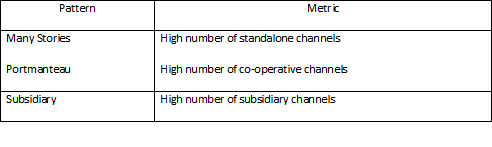

Supplement: S4 Table — (PNG) [file pone.0225910.s036.png]

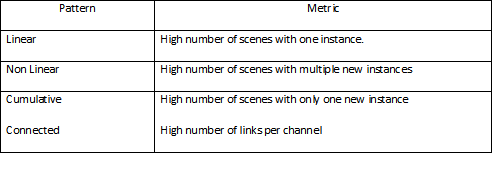

Supplement: S5 Table — (PNG) [file pone.0225910.s037.png]

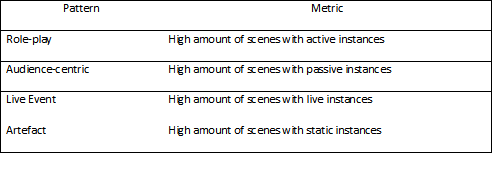

Supplement: S6 Table — (PNG) [file pone.0225910.s038.png]

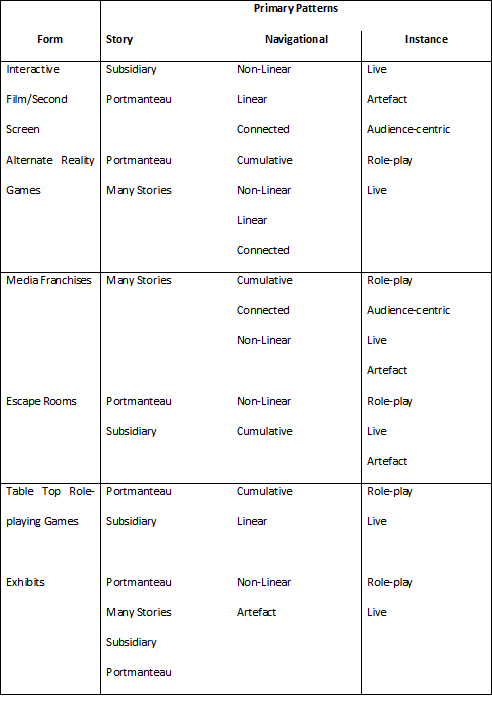

Supplement: S7 Table — (PNG) [file pone.0225910.s039.png]
