# Supplementary material for: Structural patterns for transmedia storytelling
Source: PLoS One. 2020 Jan 31;15(1):e0225910. doi: 10.1371/journal.pone.0225910 (PMC6993987; doi:10.1371/journal.pone.0225910)
Supplement: Attachment — Submitted filename: Response_to_Reviewers.docx [file pone.0225910.s040.docx]

1. Grammar - We have edited the document to fix grammar and spelling mistakes. (Reviewer 1, Reviewer 2, Reviewer 3)
2. References and Background – We have updated our background section to include more context to our research, and further related work. (Reviewer 1)
3. Signposting – We have expanded our introductory paragraph in section 5 to provide more signposting for the reader. (Reviewer 1)
4. Integrate section 6 with case studies – We felt that this would in fact make the reading much harder, and would risk talking about the same things twice. Our findings in section 6 are grouped according to the forms of the case studies. (Reviewer 1)
5. Table – We have added a table in section 6 to summarise our findings (Reviewer 3).
6. Definitions – We have included more background literature for definitions, and included our own (Reviewer 3).
7. Case study amount – We feel that we needed at least this amount to be able to identify the patterns that we did. Our objective was not to do a detailed qualitative analysis, but rather a structural analysis of how these experiences operate. (Reviewer 2)
